# A Novel Ultrasound Robot with Force/torque Measurement and Control for Safe and Efficient Scanning

**DOI:** 10.1109/TIM.2023.3239925

**Published:** 2023

**Authors:** Xianqiang Bao, Shuangyi Wang, Lingling Zheng, Richard James Housden, Joseph Hajnal, Kawal Rhode

**Affiliations:** School of Biomedical Engineering & Imaging Sciences, King’s College London, SE1 7EH, United Kingdom; State Key Laboratory of Management and Control for Complex Systems, Institute of Automation, Chinese Academy of Sciences, Beijing, 100190, China; Faculty of Engineering and Design, Kagawa University, Takamatsu 761-0396, Japan; School of Biomedical Engineering & Imaging Sciences, King’s College London, SE1 7EH, United Kingdom

**Keywords:** Ultrasound robot, robotic ultrasonography, force measurement, operation safety, ultrasound image

## Abstract

Medical ultrasound is of increasing importance in medical diagnosis and intraoperative assistance and possesses great potential advantages when integrated with robotics. However, some concerns, including the operation efficiency, operation safety, image quality, and comfort of patients, remain after introducing robotics into medical ultrasound. In this paper, an ultrasound robot integrating a force control mechanism, force/torque measurement mechanism, and online adjustment method, is proposed to overcome the current limitations. The ultrasound robot can measure operating forces and torques, provide adjustable constant operating forces, eliminate great operating forces introduced by accidental operations, and achieve various scanning depths based on clinical requirements. The proposed ultrasound robot would potentially facilitate sonographers to find the targets quickly, improve operation safety and efficiency, and decrease patients’ discomfort. Simulations and experiments were carried out to evaluate the performance of the ultrasound robot. Experimental results show that the proposed ultrasound robot is able to detect operating force in the z-direction and torques around the x- and y- directions with errors of 3.53% F.S., 6.68% F.S., and 6.11% F.S., respectively, maintain the constant operating force with errors of less than 0.57N, and achieve various scanning depths for target searching and imaging. This proposed ultrasound robot has good performance and would potentially be used in medical ultrasound.

## I Introduction

MEdical ultrasound has been playing an essential role in medical diagnosis and is expected to be a promising tool in intraoperative assistance [1–2]. During the ultrasound examination, sonographers manipulate an ultrasound probe and adjust its positions/poses, and a lengthy procedure is commonly needed to obtain desired ultrasound images. The prolonged manipulation time and repeat procedures result in a lower operation accuracy and safety and a heavy workload [3]. Due to the advantages of robotics, ultrasound robots are introduced into the field of medical ultrasound, and they can provide high manoeuvrability of the ultrasound probe, achieve long-time and stable image acquisitions, increase the scanning accuracy and safety, and reduce the sonographers’ workload [4]. In addition, ultrasound robots can be manipulated by sonographers from a distant location and this provides a possibility to carry out remote procedures [5].

Ultrasound imaging is considered one of the most operator-dependent imaging modalities [5], and thus, when ultrasound robots are used to perform scanning, the operation efficiency, operation safety, image quality, and comfort of patients are the matters to be concerned. The operation safety, image quality, and comfort of patients involve the operating force/torques. Inadequate operating forces could result in low-quality images, and great operating forces, meanwhile, may bring discomfort to patients or cause potential injuries [6–8]. Desired ultrasound images come from the ultrasound probe positioned over the desired target and with appropriate scanning depth. Determination of probe location and scanning depth determines the operation efficiency.

Researchers have been trying to measure the operating force for monitoring or as inputs for other functions. In [9], a tactile sensor was proposed for contact force measurements using piezoelectric transduction and polyvinylidene fluoride thin film. This sensor can detect forces in a range of 1 to 6 N and achieve high-resolution shape recognition. K. Wang *et al*. presented an improved force sensor by using multiple flexible thin films that can output various voltages when different forces are applied [10]. Experimental results showed the average error of the output force was less than 4.2%. Instead of designing sensors, many researchers employed commercial sensors to detect the operating force. For example, Y. Huang *et al*. [11] and C. Yang *et al*. [12] used a commercial force sensor set between a commercial robotic arm and the ultrasound probe to measure the operating force. Similarly, in [13], a commercial six-axis force/torque sensor was mounted on the end of the robotic arm to monitor the contact force of the ultrasound probe. Employing commercial force sensors can simplify the design of the ultrasound robot but 1) greatly increases the end-effector size since an additional mechanism needs to be designed to integrate the force sensor with the end-effector [14], 2) deteriorates the accuracy and affects the measurement range of the sensor due to the undesired extension of lever arms [14], and 3) extremely increases ultrasound robot cost (commercial sensor basically costs more than €4000 for 6 degrees of freedom (DOF) measurement and €1400 for 1 DOF measurement)[15]. Some other studies, such as [16], did not integrate operating force measurement into the ultrasound robot.

On the other hand, high force control accuracy results in high-quality images and safer operations and increases comfort for patients and thus many researchers have tried to control the operating force during the ultrasound examination [17]. [18] used a constant spring to keep the operating force constant. The developed robot has a simple structure, but the constant force can not be adjusted online and various springs will be needed and installed before scanning when different operating forces are required. In [19], a constant force control method was presented to maintain the applied force based on the measured force, and the error was ±50gf when the applied force was 150gf. P. Arbeille *et al*. proposed an ultrasound robot that can provide operating forces limited to 15N during the scanning [20]. However, these studies maintain the operating force by driving the robotic arm and do not separate the operating force control from the robotic arm control, which is not conducive to the control and function expansion of the robotic arm [17]. Moreover, the operating force is limited within a certain range but can not be precisely controlled and adjusted online according to the clinical requirements.

Regarding the operation efficiency, it depends on the determination of probe location and scanning depth. Many researchers have been devoted to the automatic determination of these parameters for achieving automatic scanning. R. Nakadate *et al*. presented an algorithm to automatically search and detect the common carotid artery and its tissue layers [21]. In [22], a novel dual-agent framework integrating a reinforcement learning agent and a deep learning agent, was proposed to determine the movement of the ultrasound probe based on the real-time US images. These reported studies are basically used to replace sonographers and autonomously complete the scanning, and they would have potential further medical applications. However, these methods have relatively little interaction with sonographers and are image-based rather than force-based mechanisms, thus easily introducing operation safety and patient comfort concerns [22].

Based on the above analyses, the current limitations involve multi-dimensional mechanical measurement, online adjustment and control of operating forces, and the management of scanned positions and depths of various targets according to clinical requirements, which introduce issues concerning the workload and operation efficiency of sonographers, and the comfort and operation safety of patients. In [23], Z. Cheng *et al*. proposed a method to achieve a constant output load through a positive stiffness spring and a negative stiffness elastic element. The developed manipulator can generate constant force with reasonable accuracy but does not address all the limitations above due to the lack of multi-dimensional mechanical measurement and management of scanned positions/depths of various targets. In our previous research, a customized spring-loaded ball clutch joint was proposed and integrated with the ultrasound robot to limit the maximum force applied to the ultrasound probe [24]. Meanwhile, both single-arm and dual-arm ultrasound robots were developed to perform fetal imaging [25–26], and a soft robotic end-effector was proposed to adjust the ultrasound probe position and buffer accidental operating force [7]. However, our previous solutions also failed to address these limitations discussed above.

To overcome the limitations above, in this paper, we propose a novel ultrasound robot that can measure operating force/torque, provide an online adjustable constant operating force, and change or maintain the scanning depth in real-time based on experience and clinical requirements. The main contributions of this research are as follows. 1) We propose a force/torque measurement mechanism to measure the operating forces and torques during the scanning. The measured operating forces and torques can be used as operation status monitoring, early warning, and the input of the proposed online adjustment method. 2) An online adjustment method integrated with the force/torque measurement mechanism and the force control mechanism is proposed to assist sonographers in performing ultrasound scanning. The online adjustment method can provide adjustable constant operating forces, eliminate great operating forces introduced by accidental operations, and achieve various scanning depths based on the clinical requirements, thus potentially facilitating sonographers to find the targets quickly, improving operation safety and efficiency, and decreasing patients’ discomfort.

The remainder of this paper is presented as follows. Section II details the proposed methods, including the force control mechanism, force/torque measurement mechanism, online adjustment method, prototype, and other details. In Section III, simulations and experiments evaluating the performance of the proposed ultrasound robot are reported. Conclusions and future work of this research are given in Section IV.

## II Methodology

### A System Description

**Fig. 1:**
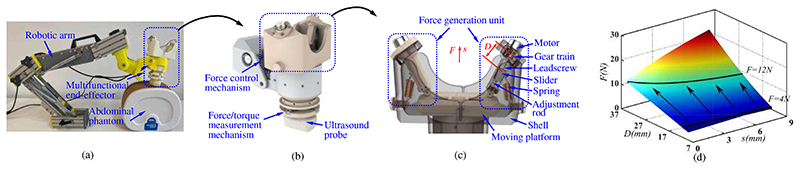
Proposed ultrasound robot: (a) prototype; (b) multifunctional end-effector; (c) force control mechanism; (d) constant force generation and regulation diagram (CFGRD).

The proposed ultrasound robot, as shown in Fig. 1 (a), consists of a robotic arm and a multifunctional end-effector. The multifunctional end-effector is mounted on the end of the robotic arm that can adjust positions through the rotation of each joint with the control of sonographers. The multifunctional end-effector grasps an ultrasound probe and moves along the abdomen to acquire ultrasound images. As shown in Fig.1 (b), the multifunctional end-effector has a force control mechanism and force/torque measurement mechanism that enables the ultrasound probe to perform the scanning with constant forces and measures the operating forces/torques during the scanning, respectively. With the constant operating force, ultrasonography could be performed safely, and discomfort for patients would also be decreased. The measured forces and torques can be used as operation status monitoring and early warning during scanning procedures. This multifunctional end-effector is independent of the robotic arm and performs autonomous control, which is conducive to expanding other functions of the robotic arm.

### B Force Control Mechanism

As shown in Fig. 1 (c), the force control mechanism is composed of two force generation units that are connected in parallel and linked with the moving platform. The force generation unit comprises a motor, gear train, leadscrew, slider, spring and adjustment rod. The motor, gear train, leadscrew, and slider are installed in the adjustment rod that links the moving platform and the shell of the multifunctional end-effector. The motor drives the leadscrew through the gear train, and the slider will have various positions relative to the leadscrew when the leadscrew rotates. The spring links the slider and the shell, and its deformation changes with the movement of the slider. Different generated forces will be generated by various deformations of the spring, and thus, the motor can precisely control the generated force according to the position of the slider (*D*) and the displacement of the moving platform (*s*). The rotation of the motor will determine the D and a displacement measurement unit set under the moving platform can measure the *s*. Fig. 1 (d) shows the constant force generation and regulation diagram (CFGRD) that describes the relationship between these parameters. This force control mechanism can produce constant forces from 4 N to 12 N. Some analysis can be seen in [17].

### C Force/torque Measurement Mechanism

#### 1) Structure

**Fig. 2:**
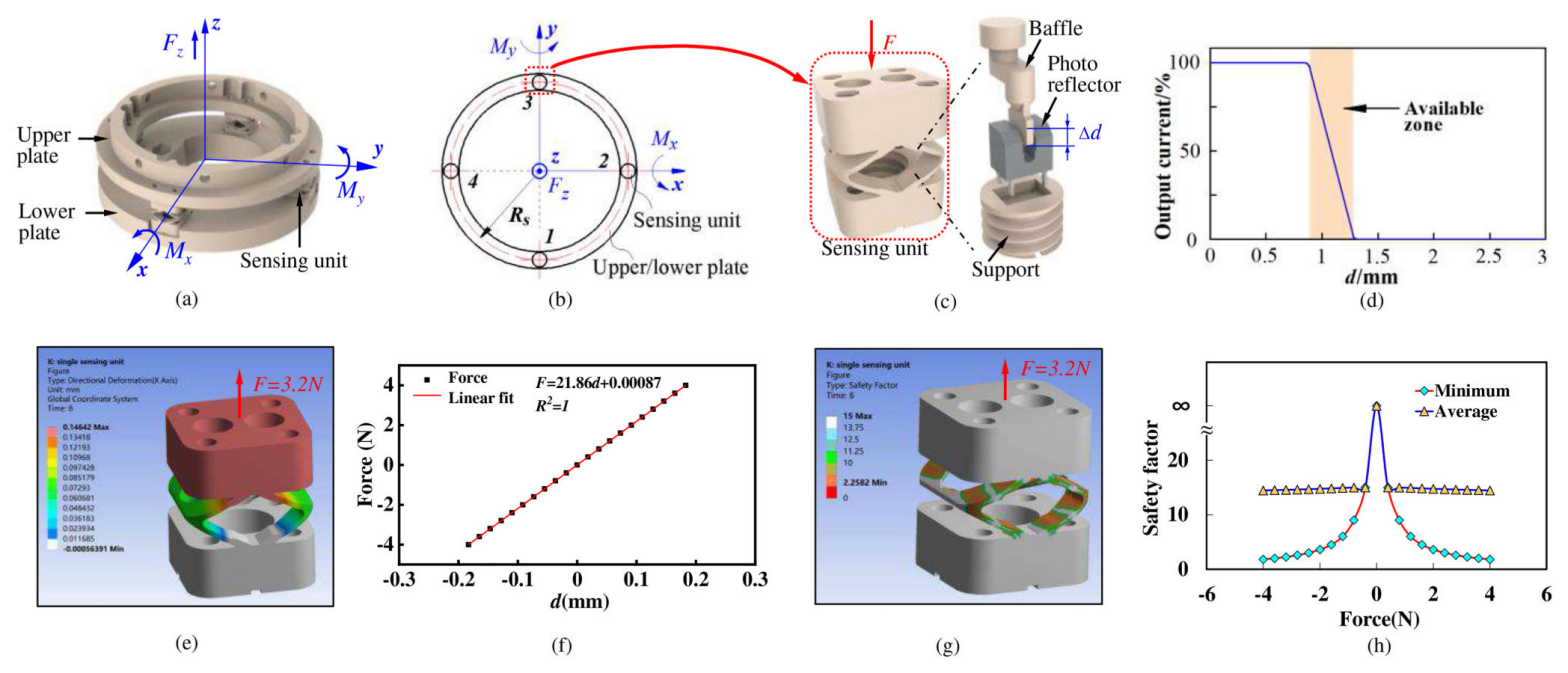
Force/torque measurement mechanism: (a) structure diagram; (b) force analysis diagram; (c) sensing unit; (d) the relationship between the measured displacement and output of the photo reflector; (e) directional deformation (along the force direction) of the sensing unit with force applied; (f) the relationship between the applied force and directional deformation of the sensing unit; (g) safety factor for the sensing unit with force applied; (h) minimum and average safety factors when various forces applied.

To measure the operating force and torque during the scanning, a force/torque measurement mechanism is developed and integrated into the multifunctional end-effector (Fig. 1 (b)). As shown in Fig. 2 (a) and (b), the force/torque measurement mechanism consists of an upper plate, four sensing units and a lower plate. The four sensing units are evenly arranged in a ring and connect the upper plate and the lower plate. When forces and torques are applied to the sensing unit, the sensing unit deforms and the baffle moves with deformations (Fig. 2 (c)). A photo reflector (RPI-121, ROHM, JP) set in the support is used to detect the movement of the baffle (Δ*d*). This photo reflector is composed of a LED and photo-transistor. When the baffle moves with various displacements, the photo-transistor obtains infrared signals with varying light intensity from the LED. With the light intensity variation, the photo reflector will output various voltages. Thus, the deformation of the sensing unit can be calculated through the output voltages of the photo reflector. Fig. 2 (d) shows the relationship between the measured displacement and the output of the photo reflector. The available zone of this photo reflector is highlighted in orange and the measurement range is about 0.34mm.

#### 2) Simulation

In order to investigate the structural parameters and quantify the performance of the sensing unit, simulations were carried out based on the Finite Element Method (FEM). Since the prototype will be fabricated through 3D printing, the model analyzed in the ANSYS is given the same properties as the 3D printing material, i.e., Young’s modulus of 1800MPa and Poisson ratio of 0.35. We first applied forces of ±3.2N to the sensing unit and observed its largest directional deformation (along the force direction). The simulation results are shown in Fig. 2 (e). The largest deformations under the forces of 3.2N and -3.2N are 0.14642mm and 0.14643mm, respectively. Since these two cases have similar results, we only show one of them. The total deformation of the sensing unit is 0.293mm, and this is in the measurement range of the photo reflector (0.34mm), which means the required deformation can be measured by the photo reflector.

In addition, we applied a progressively increasing force with 0.4-N increments from -4 N to 4N to the sensing unit. Fig. 2 (f) shows the relationship between the applied force and the deformation of the sensing unit. The applied force has a linear relationship (R^2^=1) with the deformation, and thus the sensing unit will have good linearity for force measurement. The reliability of a design can be described by safety factors, and normally, a safety factor greater than 2 indicates good reliability [27]. We exerted various forces from -4 N to 4N on the sensing unit and the simulation results are shown in Fig. 2 (g) and (h). The structures with lower safety factors are mainly distributed on the beams. A greater applied force results in a smaller safety factor, and the minimum safety factor is 2.26 when forces of ±3.2N are applied. Therefore, the proposed sensing unit will have good reliability for the force measurement.

#### 3) Modelling and force analysis

When the multifunctional end-effector grasps the ultrasound probe and moves along the abdomen, the force/torque measurement mechanism is subjected to operating forces and torques, and each sensing unit will be deformed with applied forces *F_i_*. The *i* indicates the serial number of the sensing units, and its value can be taken as 1, 2, 3, and 4. Based on Fig. 2 (a) and (b), the operating forces and torques can be written by

(1) $$\left[\begin{array}{c} F_{z}\\ M_{x}\\ M_{y} \end{array}\right]=\left[\begin{array}{c} \sum F_{i}\\ R_{s}F_{3}-R_{s}F_{1}\\ R_{s}F_{4}-R_{s}F_{2} \end{array}\right]$$

where *F_z_*, is the force exerted on the force/torque measurement mechanism in the *z*-direction, *M_x_* means the torque applied to the force/torque measurement mechanism around the *x*-direction, *M_y_* means the torque applied to the force/torque measurement mechanism around the *y*-direction, and *R_s_* is the radius of the distribution circle of sensing units. As shown in the simulation results (Fig. 2 (f)), the deformation of the sensing unit has a linear relationship with the applied force, and thus there will be

(2) $$F_{i}=k\delta_{i}$$

where *k* means the stiffness of the sensing unit and *δ_i_* is the deformation of the sensing unit *i*. In addition, as shown in Fig. 2 (d), the photo reflector outputs voltages that scale linearly with the distance in the available zone, and thus the deformation of the sensing unit can be obtained by

(3) $$\delta_{i}=\alpha \Delta U_{i}$$

where *α* means the output coefficient of the photo reflector and *U_i_* indicates the output voltages of the photo reflector in sensing unit *i*.

To facilitate calculation and analysis, we transformed the four voltage parameters from the sensing units into three ones that directly correspond to the measured forces/torque. The transformation is expressed as

(4) $$\left[\begin{array}{c} \Delta U_{z}\\ \Delta U_{x}\\ \Delta U_{y} \end{array}\right]=\left[\begin{array}{c} \Delta U_{1}+\Delta U_{2}+\Delta U_{3}+\Delta U_{4}\\ \Delta U_{3}-\Delta U_{1}\\ \Delta U_{4}-\Delta U_{2} \end{array}\right]$$

where *U_z_*, *U_x_*, and *U_y_* are the equivalent output voltages for force and torque measurement in/around the *z*-, *x*-, and *y*- directions, respectively, and *U*_*i*|*i*=1,2,3,4_ mean the output voltages of the photo reflector in sensing unit *i*. Substituting equations (2)−(4) into (1) results in

(5) $$\left[\begin{array}{c} F_{z}\\ M_{x}\\ M_{y} \end{array}\right]=\left[\begin{array}{ccc} k\alpha & 0 & 0\\ 0 & R_{s}k\alpha & 0\\ 0 & 0 & R_{s}k\alpha \end{array}\right]\left[\begin{array}{c} \Delta U_{z}\\ \Delta U_{x}\\ \Delta U_{y} \end{array}\right].$$

Based on equation (5), the operating forces and torques during the ultrasound scanning can be obtained.

### D Online Adjustment Method

Ultrasound acquisitions require substantial experience and training of sonographers, and especially for images of different cross-sections of the same organ or tissue, sonographers need to control the operating force precisely to obtain various scanning depths, which involves operation safety and efficiency.

**Fig. 3:**
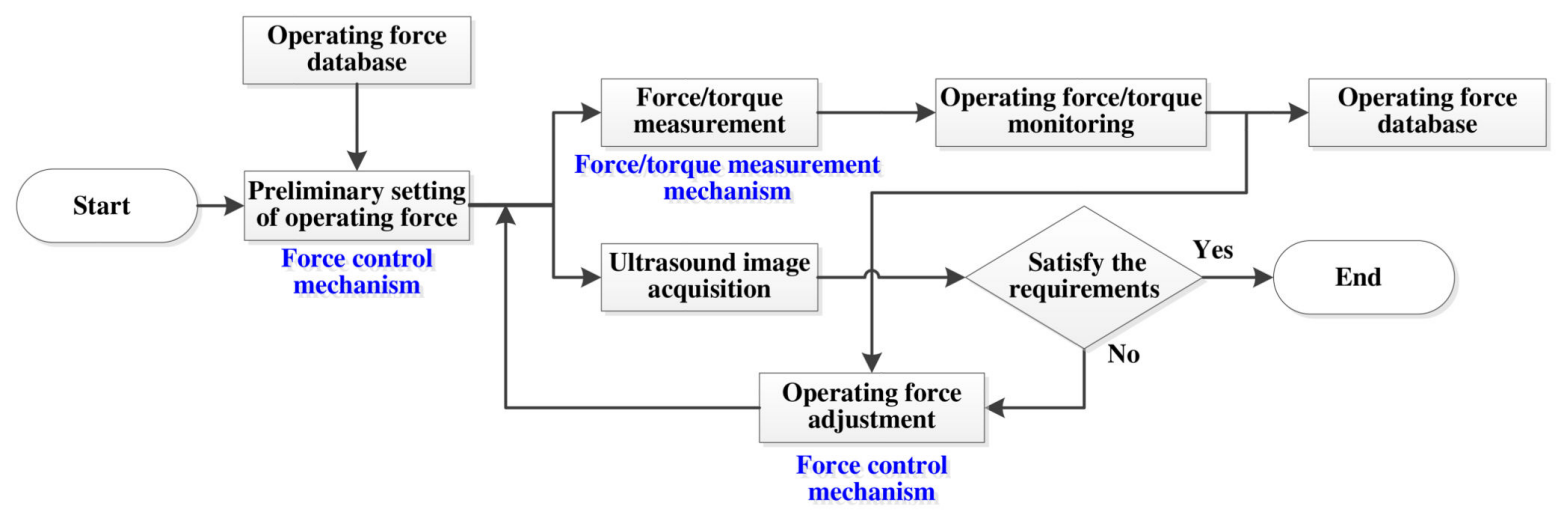
Block diagram of online adjustment method.

In order to improve scanning safety and efficiency, a novel online adjustment method is proposed. The online adjustment method integrates the force control mechanism and force/torque measurement mechanism. Fig. 3 shows the block diagram of the proposed online adjustment method. When the ultrasound robot starts to work, the preliminary operating force will be set to obtain a potentially suitable scanning depth based on the operating force database. The operating force database is built according to the previous operating force data and the sonographers’ professional experience. The force control mechanism is set to have a specific control parameter D (Fig. 1 (d)) and produces constant operating forces. Then, the ultrasound robot acquires ultrasound images with the set scanning depth/operating force. During the image acquisition, the force/torque measurement mechanism measures the operating forces and torques and uses them as operation status monitoring and early warning. Also, these operating forces and torques are stored in the operating force database. Meanwhile, various ultrasound images are obtained, and sonographers observe the scanned area and make a diagnosis. If the acquired images satisfy the requirements for diagnosis, this scanning procedure is completed; if not, the control parameter of the force control mechanism will be reset to generate operating forces of different values based on the operating force database, and then various scanning depths will be obtained for further scanning.

**Fig. 4:**
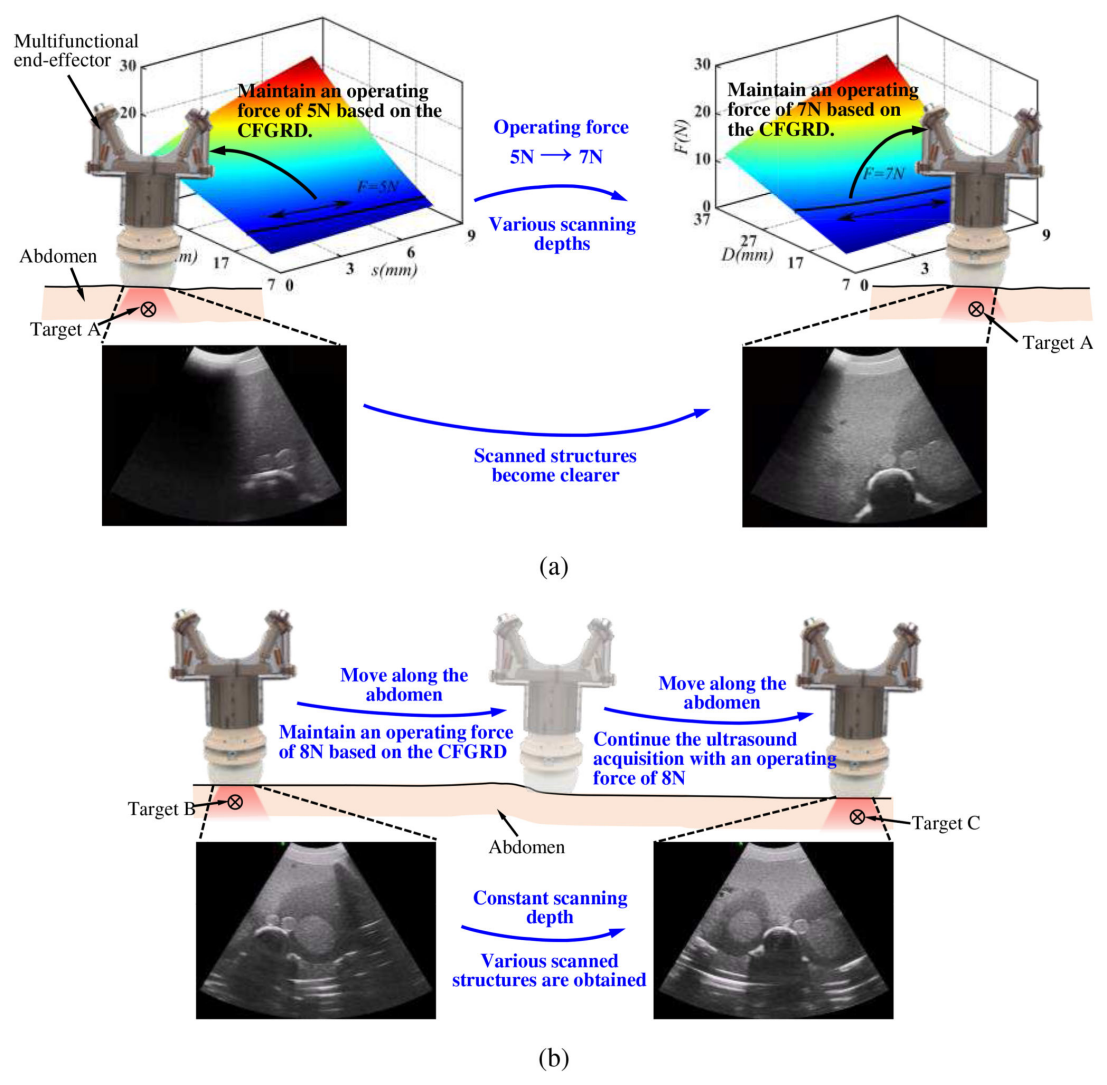
Specific operation process of the online adjustment method for scannings with one target (a) and with various targets (b).

Fig. 4 (a) and (b) show the specific operation process. When the ultrasound probe is positioned above the target area, to acquire the ultrasound images of target organs or tissues, it needs to adjust the operating force to obtain various scanning depths. As shown in Fig. 4 (a), the ultrasound probe is positioned above target A, and to achieve a suitable scanning depth, the ultrasound robot produces a constant operating force of 5N based on the operating force database and CFGRD. Ultrasound images are then obtained, but they are not clear enough to make a diagnosis. The ultrasound robot then adjusts the scanning depth by changing the operating force to 7N and keeping it constant during the operation. By increasing the operating force, the scanning depth changes and more clear images are acquired. During this scanning, sonographers don’t have to care about operation safety and the patient will not receive discomforts introduced by undesired or great forces. In addition, various operating forces result in different scanning depths, and the scanning depth will be determined and not change with the operation when the operating force is set as a constant value, therefore facilitating finding the target easily and quickly and significantly improving the operation efficiency.

Commonly, different organs or tissues are required to be scanned after images of an area are acquired, and the ultrasound probe needs to keep sliding along the abdomen to search for other targets. During the probe movement, the scanning depth changes in real time. For similar structures, such as a cross-section of the left and right sides of the kidney, the required scanning depths will be approximately equal. Therefore, the scanning depth can be maintained with the same value to carry out the target search and complete the scanning. As shown in Fig. 4 (b), after achieving the ultrasound images of target B, target C needs to be searched and scanned. Since target C has a similar scanning depth to target B, the ultrasound robot is still set to generate an operating force of 8N and manipulates the ultrasound probe to move along the abdomen for target searching. On the other hand, for the targets with various scanning depths, we can use the operating force database as a reference to complete the setting of the operating force. Certainly, when target C is found but can not be scanned clearly with the operating force of 8N, a similar process to Fig. 4 (a) could be performed to fine-tune the scanning depth until a clearer image is obtained. Similar to the process in Fig. 4 (a), this process can also improve operation safety and efficiency and bring decreased discomfort to patients.

### E Prototype and Other Details

**Fig. 5:**
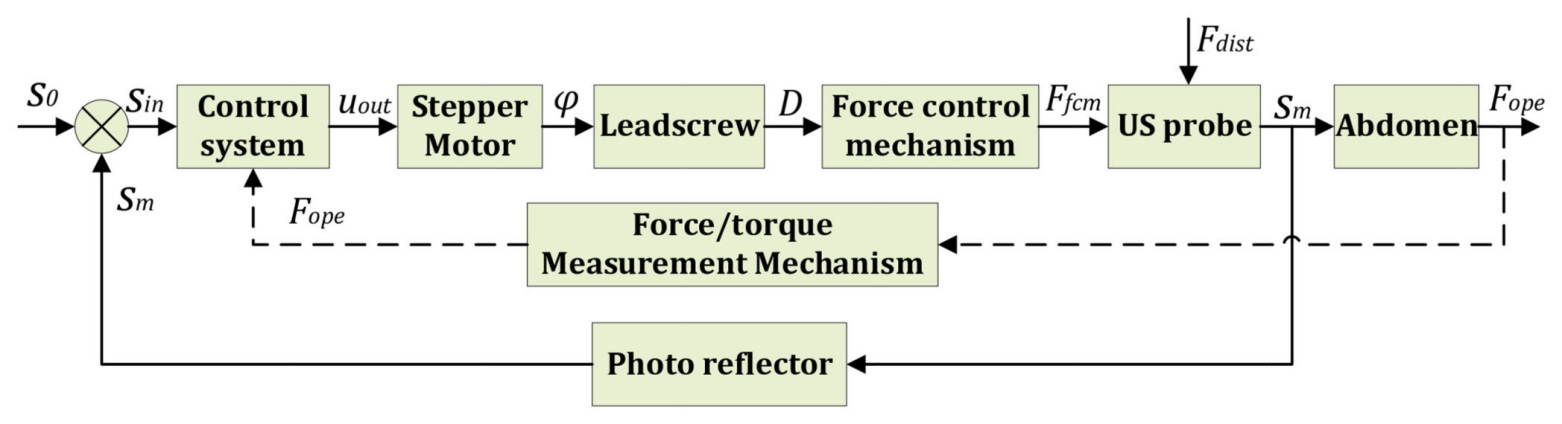
Control block diagram for force adjustment of the US probe.

The ultrasound robot has dimensions of 820 mm × 155 mm ×450 mm and can provide manipulations with 8 DOFs. The ultrasound robot was assembled in the laboratory in which most of the robotic arm was fabricated from aluminium alloy, the multifunctional end-effector was 3D printed, and some key components, such as stepper motor (HBD12-D, MCRT, CN), tension spring (DE675, Accurate, JP), rail kit (BSP1025SL, IKO, JP), bearing (C-LMUM3, MISUMI, JP), motor drivers (TB6600 upgraded, JXINW, CN), data acquisition module (DAM-3918, ART, CN), photo reflector (NJL5909RL-4, JRC, JP), and photo reflector (RPI-121, ROHM, JP), were purchased from suppliers. To enable the ultrasound robot to perform the online adjustment method smoothly, we used a force adjustment method to control the US probe. As shown in Fig. 5, the control system outputs signals (*u_out_*) to control the rotations of the stepper motor (*φ*) based on the input signals (*s_in_*) that are measured displacements of the US probe by the photo reflector mounted on the moving platform (*s_m_*) subtracted from the original displacement for the desired output force (*s*_0_). The position of the slider (*D*) determines the output force of the force control mechanism (*F_fcm_*) (see Fig. 1 (d)). During the scanning, the US probe will be applied with disturbance force (*F_dist_*), and the resultant operating force (*F_ope_*) is captured by the force/torque measurement mechanism and sent to the control system as operation status monitoring, early warning, and the input of the proposed online adjustment method.

The ultrasound probe is grasped by the lower plate in the force/torque measurement mechanism (see Fig. 2 (a)), and various lower plates are needed for different types of ultrasound probes because different ultrasound probes have diverse shapes and dimensions. Hence, ideally, each ultrasound probe is equipped with a multifunctional end-effector that can be quickly installed on or removed from the robotic arm. However, if there is only one multi-functional end-effector due to limited conditions, we can replace the lower plate for grasping various ultrasound probes. This replacement will result in changes in the centre of gravity of the multifunctional end-effector, and thus, compensation will be needed for force measurement.

## III Experimental Verification

### A Simulations

#### 1) Experimental setup

To test the performance of the proposed force/torque measurement mechanism, FEM-based simulations were carried out by using ANSYS. The simulations have the same material settings as those in Section II-C. The response performance for force measurement was evaluated by applying forces in the *z*-direction with 2-N increments from -12 N to 12N to the upper plate of the force/torque measurement mechanism. Similarly, torques around both *x*- and *y*-directions from -180 N·mm to 180 N·mm with 30-mN·m increments were also exerted on the upper plate. Meanwhile, the lower plate of the force/torque measurement was firmly connected to the ground.

To test the performance of the ultrasound robot applied with disturbance, we conducted simulations by exerting various disturbance forces on the US probe and observed the responses. The simulations were carried out via the Simulink module in MATLAB, which is always used to simulate the dynamic response of motion systems [28–29]. The ultrasound robot generates forces based on the equation $F=2k\frac{L_{1}L_{2}}{L_{2}}+\frac{2L_{1}L_{3}}{L_{3}}\cdot \frac{F_{0}-kL_{0}}{\sqrt{L_{0}^{2}+2L_{1}L_{3}s/L_{2}}}$ [17], and the corresponding and other necessary parameters, including equivalent mass (*m*) and equivalent damping coefficient (*b*), were set as follows: *k*=1.236 N/mm, 9mm⩽*L*_1_⩽35mm, *L*_2_=45mm, *L*_3_=40mm, *F*_0_=6.257 N, *L*_0_=31.9 mm, m=0.9kg, and b=1Ns/m. The desired operating force was set to 8N and the initial position of the US probe was 0mm. Two types of forces, ramp signals (slope: ±3.75m/s, maximum value: 3N, see Fig. 7 (a)) and sine signals (*y*=2sin5*t*, see Fig. 7 (d)), were employed to simulate the disturbances and exerted on the US probe.

#### 2) Results and discussions

**Fig. 6:**
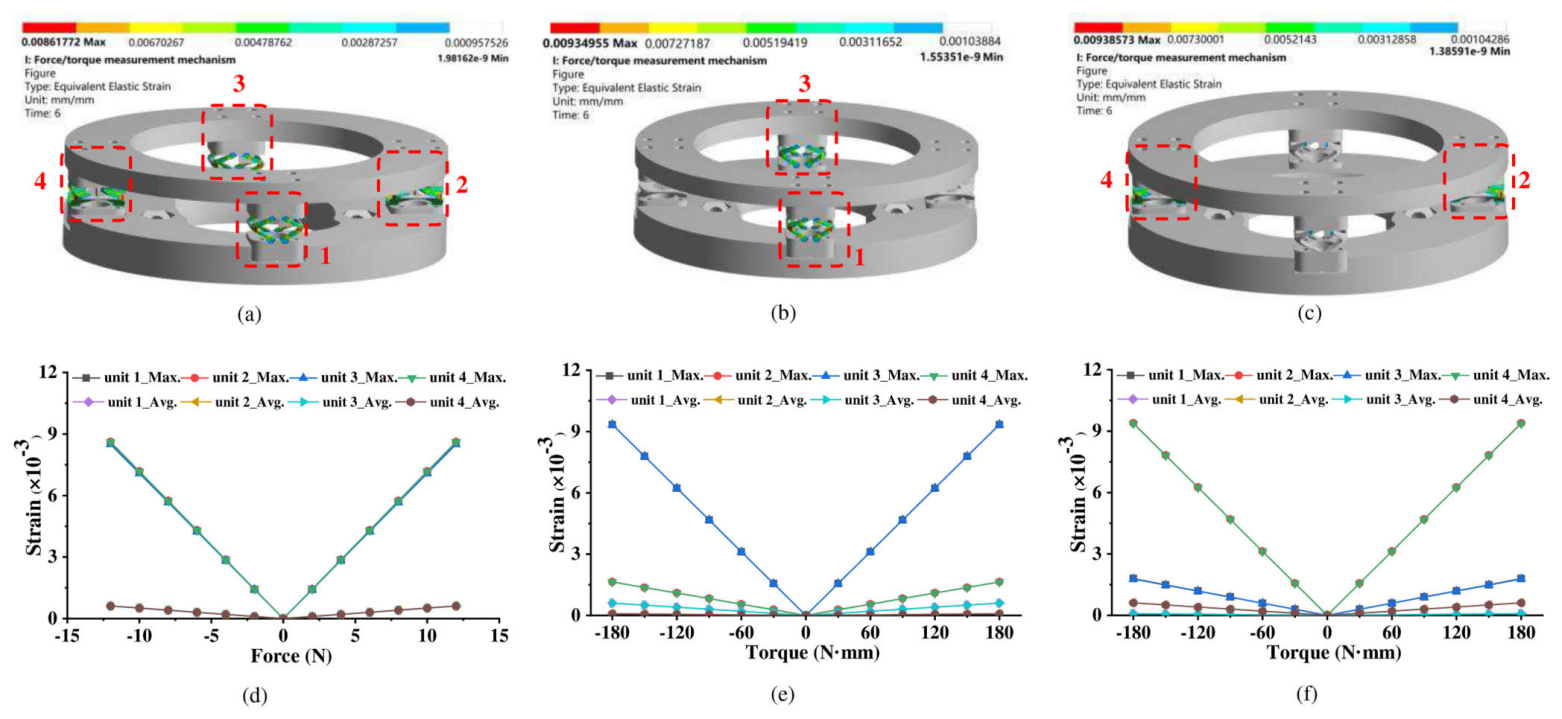
Simulation results for the force/torque measurement mechanism. Strain distribution diagrams under 12N in the *z*-direction (a), 180 N·mm around the *x*-direction (b), and 180 N·mm around the *y*-direction (c). Maximum and average strain of each sensing unit with various applied forces/torques: forces in the *z*-direction (d), torques around the *x*-direction (e), and torques around the *y*-direction (f).

Fig. 6 shows the strain distribution and variation of the proposed force/torque measurement mechanism. As indicated in Fig. 6 (a) and (d), the strain occurs in all four sensing units and has a highly linear relationship with applied force in the *z*-direction. In addition, the maximum and average strain in these four sensing units have almost the same values. With the applied torques around the *x*-direction, sensing units 1 and 3 have almost the same strain with large values (Fig. 6 (b) and (e)). Tiny strain also occurs in both sensing units 2 and 4. Similar situations remain in Fig. 6 (c) and (f) when torques around the *y*-direction are applied. Based on the simulation results, we can find that 1) all the strain has a highly linear relationship with applied forces and torques, 2) all the four sensing units possess the same great strain with forces applied, 3) two sensing units possess the same great strain while the other two remain the same small strain with torques exerted. Therefore, it will be a good choice to build a formula Δ*U*_1_ + Δ*U*_2_ + Δ*U*_3_ + Δ*U*_4_ to calculate the force in the *z*-direction, and build formulas Δ*U*_3_ − Δ*U*_4_ and Δ*U*_4_ − Δ*U*_2_ to estimate the torques around the *x*- and *v*-direction, respectively. This method is consistent with equations (4) and (5) above. The FEM-based simulation also potentially indicates that the proposed force/torque measurement mechanism possesses good sensing performance, such as high linearity and good sensitivity. The further characteristics of the proposed force/torque measurement mechanism will be tested in Section III-B.

**Fig. 7:**
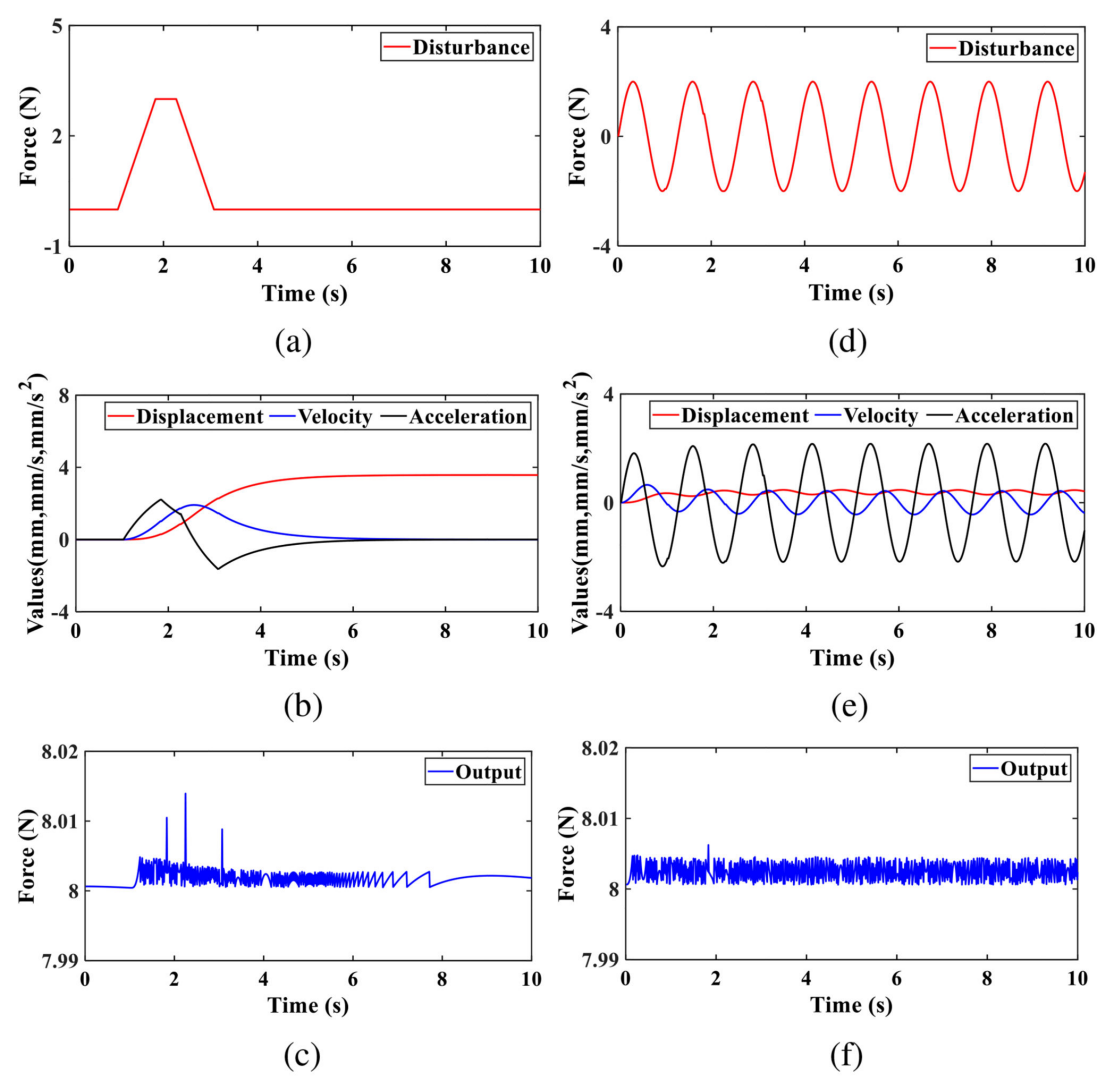
Simulation results for the disturbance. Response under slope disturbance: (a) applied disturbance force; (b) displacement, velocity, and acceleration of the US probe; (c) output force of the robot. Response under sine disturbance: (d) applied sine force; (e) displacement, velocity, and acceleration of the US probe; (f) output force of the robot.

Fig. 7 shows the simulation results under two types of disturbances, and it includes the applied forces, displacements, velocities, and accelerations of the US probe, and the output forces of the ultrasound robot. In the case of ramp disturbances, the displacement of the US probe increased when the ramp forces were applied, and it finally reached and kept 3.57mm after the ramp forces disappeared (Fig. 7 (b)). The velocities and accelerations of the US probe varied accordingly during this process. To enable the ultrasound robot to eliminate the effect of the disturbances, the control system sent out real-time control signals according to the displacements of the US probe to adjust the force control mechanism (Fig. 5), and therefore, the output forces were maintained to the desired values, i.e., 8N, and the theoretical maximum error was less than 0.02N (Fig. 7 (c)). In the case of sine disturbances, the displacements, velocities, and accelerations of the US probe kept changing during the process since the sine disturbances varied continuously, and they had the same frequencies as the sine disturbances (Fig.7 (e)). The amplitudes of the displacement, velocity, and acceleration of the US probe were 0.087mm, 0.431mm/s, and 2.169mm/s^2^, respectively. The output forces were also maintained at the desired values with the theoretical maximum error of less than 0.01N (Fig. 7 (f)). However, in the actual application, the response will be affected by various factors, such as the measurement accuracy of displacement, response time of the control system, positioning accuracy of the stepper motor, the backlash of mechanisms, etc., and thus the accuracy of output force control will inevitably decrease. To further investigate the performance, phantom experiments were carried out in Section III-D.

### B Calibration Experiments

#### 1) Experimental setup

**Fig. 8:**
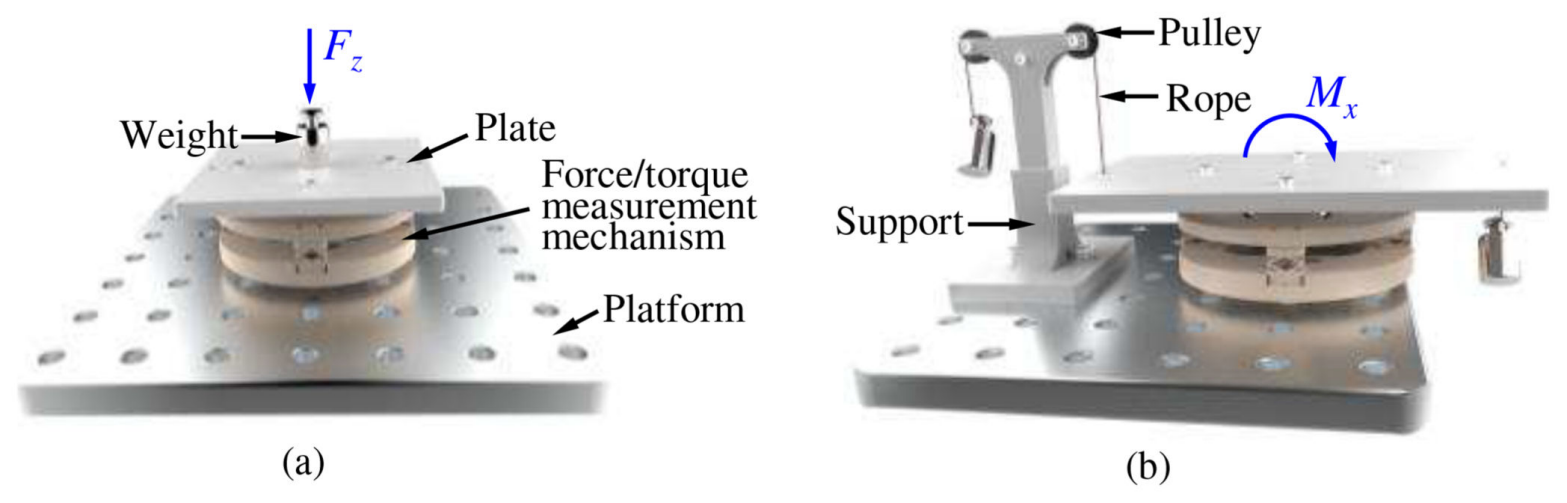
Experimental setup for calibrations of the force measurement in the *z*-direction (a) and torque measurement around the *x*-direction (b).

In the calibration experiments, various weights were used to generate different forces and torques, and the setup is shown in Fig. 8. The force/torque measurement mechanism mounted on a platform is linked to a plate that can transmit generated forces and torques. Forces in the -*z*-direction are produced when weights are set on the plate. As shown in Fig. 8 (b), two pulleys are installed in a support fixed on the platform. Two weights are joined to the plate by ropes, and thus various torques can be produced and exerted on the force/torque measurement mechanism. The forces in the +*z*-direction and torques around the ±*x*- and ±*y*-direction can be obtained through the setup in Fig. 8 (b). In the force measurement calibration experiments, we applied forces in the *z*-direction with 2-N increments from - 12 N to 12N to the force/torque measurement mechanism, and the output voltages were recorded accordingly. Each procedure was repeated six times. Similarly, torques around both *x*- and *y*-directions from -168 N·mm to 168 N·mm with 28-N·mm increments were also generated for the torque measurement calibration.

#### 2) Results and discussions

**Fig. 9:**
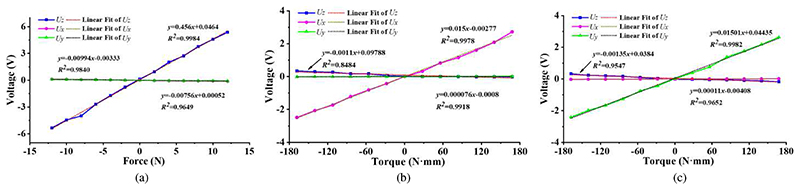
Calibration results with various forces and torques applied: (a) forces in the *z*-direction; (b) torques around the *x*-direction; (c) torques around the *y*-direction.

In our design, the end of the baffle is located in the middle position of the photo reflector, and the photo reflector outputs a voltage of 2.275V when no forces and torques are exerted on the force/torque measurement mechanism. To obtain a calculation model that is closer to a proportional relationship rather than just a linear one, the output voltages of all four sensing units were subtracted by 2.275V. The collected voltages and corresponding forces/torques were processed based on equation (4) and are shown in Fig. 9. The relationship between the voltages and forces/torques can be expressed as

(6) ΔU=AG+B

(7) $$A=\left[\begin{array}{ccc} 0.456 & -0.0011 & -0.00135\\ -0.00994 & 0.015 & 0.00011\\ -0.0076 & 0.000076 & 0.01501 \end{array}\right]$$

(8) $$B=\left[\begin{array}{c} 0.18268\\ -0.01015\\ 0.04407 \end{array}\right]$$

where Δ*U* means the voltage variation, Δ*U* = [Δ*U_z_* Δ*U_x_* Δ*U_y_*]^*T*^, ***A*** is the slope matrix, ***G*** indicates the applied forces and torques, ***G*** = [*F_z_* *M_x_* *M_y_*]^*T*^, and ***B*** is the intercept matrix. After investigating the impact of the intercept matrix on the calculation results, we found that little effect would be produced. Hence we removed the intercept matrix from equation (6) and there is

(9) $$G=M_{c}\Delta U$$

(10) $$M_{c}=\left[\begin{array}{ccc} 2.1998 & 0.1603 & 0.1967\\ 1.4496 & 66.7748 & -0.3590\\ 1.1065 & -0.2569 & 66.7236 \end{array}\right]$$

where ***M***_*c*_ is the transformation matrix for the force and torque measurement. In addition, the numerical stability of the transformation matrix can be characterized through the matrix condition number. The greater condition number indicates a more singular matrix, and normally, a matrix becomes ill-conditioned when the condition number is larger than 1000. The ill-conditioned matrix is very sensitive to input, and with the ill-conditioned matrix, small noise will lead to drastic changes in measured force and torque. The condition number can be calculated through

(11) $$\kappa_{c}=\left\|M_{c}\right\|\cdot \left\|M_{c}^{-1}\right\|.$$

Based on equations (10) and (11), the condition number of the transformation matrix is 30.59, which means this calibration is well-conditioned. Thus, the proposed force/torque measurement mechanism can measure the operating forces and torques by equations (9) and (10). More characteristics of the force/torque measurement mechanism are shown in TABLE I.

When an ultrasound probe is installed into the multifunctional end-effector, the weight of the ultrasound probe will affect the force measurement, and it needs to be compensated during the scanning. This compensation mainly depends on determining the angle between the probe axis and the gravity direction. There are two methods for the determination of the angle. (1) The angle can be calculated through the motions of the robotic arm. (2) Before the probe contacts the scanned area, the bottom surface of the probe is set parallel to the scanned area with no contact, and then the angle can be calculated by the forces and torques measured by the force/torque measurement mechanism. The first method is relatively simple and can be applied to any contour scanning area, but it totally depends on the precise data of each joint angle of the robotic arm. The second method can work independently without relying on the robotic arm, but the operating process is relatively cumbersome, and it is only suitable for occasions where the slope of the scanned area changes little.

### C Force and Torque Measurement

#### 1) Experimental setup

**Fig. 10:**
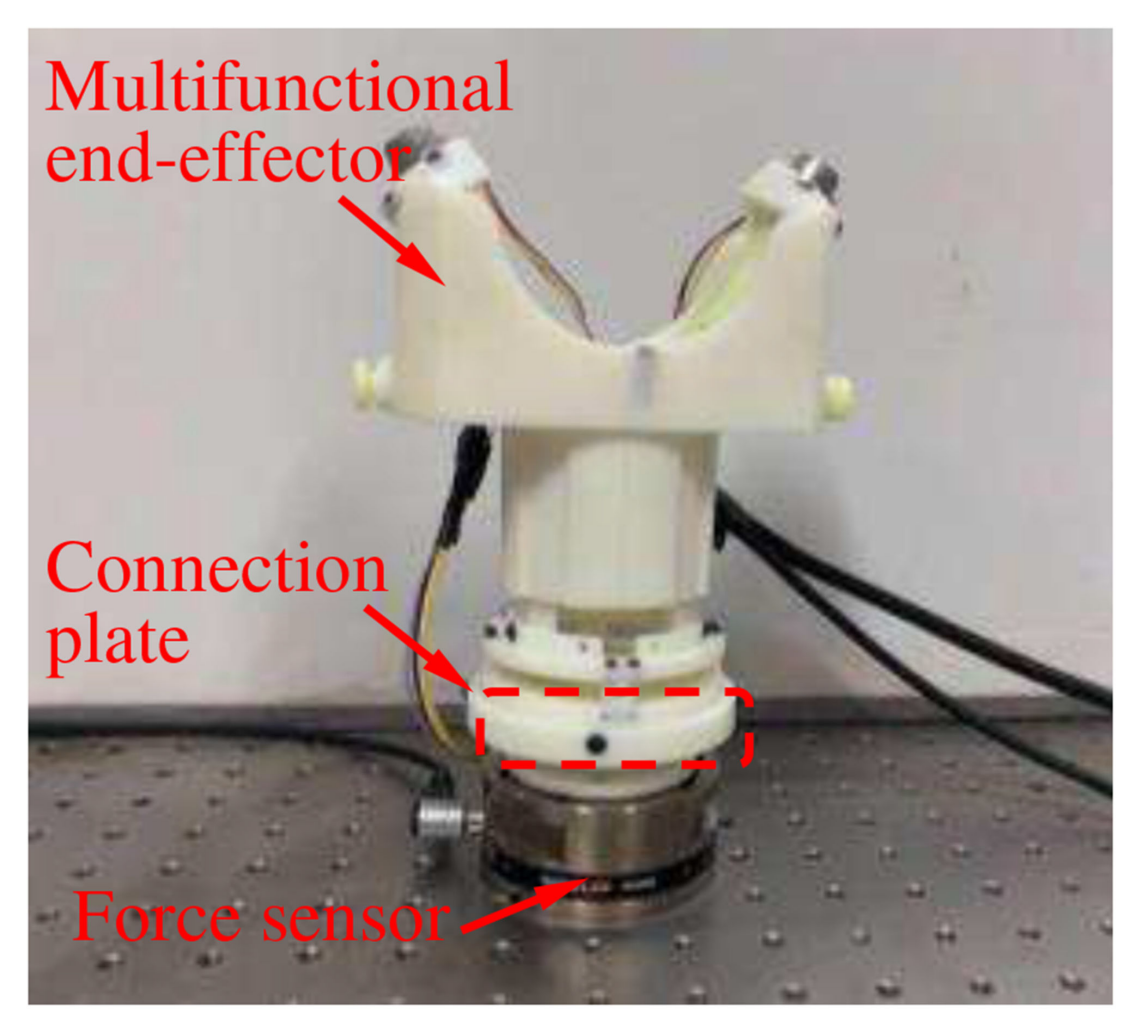
Experimental setup for force and torque measurement.

In order to evaluate whether the proposed force/torque measurement mechanism can measure the operating forces and torques during the scanning, experiments were carried out by manipulating the multifunctional end-effector randomly to simulate the possible scanning operation. The experimental setup is shown in Fig. 10. A six-axis force sensor (Gamma, ATI Industrial Automation, Inc., USA) mounted on an optical platform is linked to the force/torque measurement mechanism through a connection plate. It can measure forces and torques in/around all directions. When we operated the multifunctional end-effector, the forces and torques were captured by both the force sensor and the force/torque measurement mechanism.

#### 2) Results and discussions

**Fig. 11:**
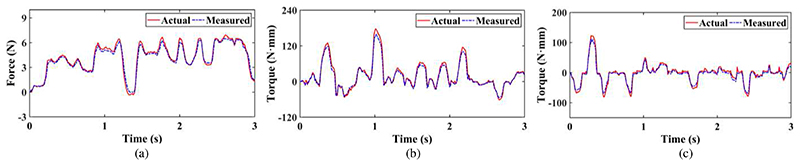
Force and torque measurement results: (a) force in the *z*-direction; (b) torques around the *x*-direction; (c) torques around the *y*-direction. The actual forces/torques mean the data recorded by the commercial sensor, and the measured forces/torques represent the data captured by our proposed force/torque measurement mechanism.

Fig. 11 shows the measured forces and torques in the experiments. The maximum errors in the measurement of forces in the *z*-direction, torques around the *x*-direction, and torques around the *v*-direction are 0.846 N, 22.45 N·mm, and 20.52 N·mm, respectively. The relative errors in the three directions are 3.53% F.S., 6.68% F.S., and 6.11% F.S., respectively. Great errors generally occur with rapid changes in forces and torques. For example, the force decreased sharply at about 1.3s and a great force measurement error of 0.846N occurred at this time. In this research, to facilitate the performance test of the proof-of-concept design, the force/torque measurement mechanism was fabricated through 3D printing. The uniformity and stability of the material composition, and dimension and installation accuracy of printed components are inevitably affected by 3D printing, which will decrease the measurement accuracy. In practical applications, the measurement accuracy of our proposed force/torque measurement mechanism can be improved to some extent by changing the manufacturing methods and materials, such as employing high-precision processing and materials with small hysteresis. Hence, 3D printing or other manufacturing methods or materials can be selected to fabricate the force/torque measurement mechanism according to the accuracy requirements. These experiments show the ability of the force/torque measurement mechanism to measure the operating forces and torques of the ultrasound probe that can be used as operation status monitoring, early warning, as well as control parameters of the proposed online adjustment method.

### D Phantom Experiments

#### 1) Experimental setup

**Fig. 12:**
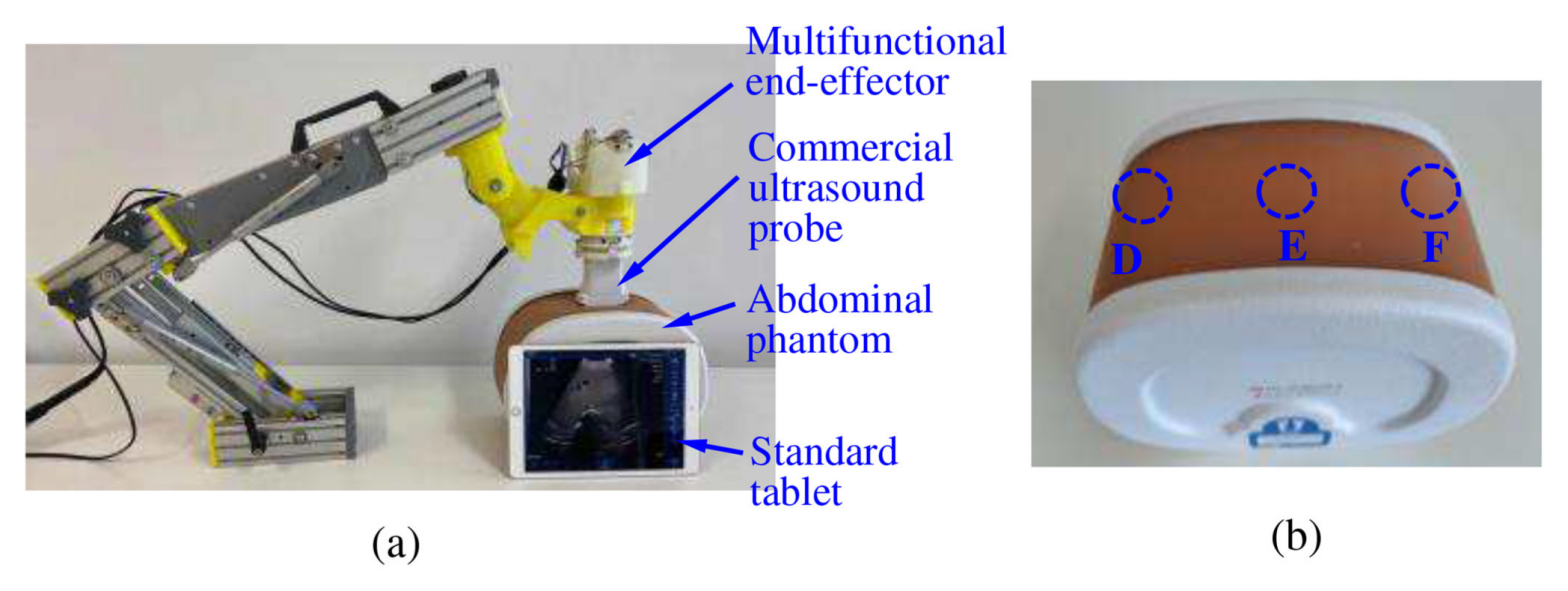
Experimental setup for phantom experiments (a) and the abdominal phantom with three marked regions (b).

To test whether the ultrasound robot can perform the ultrasound scanning using the proposed online adjustment method, phantom experiments were conducted, and the experimental setup is shown in Fig. 12. Since the proposed ultrasound robot integrates the force control mechanism, force/torque measurement mechanism, and online adjustment method, the performance of the operating force control and operating force/torque measurement can also be observed in these experiments. As shown in Fig. 12 (a), a wireless ultrasound probe (CProbe, Sonostar Technologies Co., Ltd., CN) was installed in the multifunctional end-effector, and it could move along a commercial abdominal phantom (057A, Computerized Imaging Reference Systems, Inc., USA). Images acquired by the ultrasound probe were transmitted to a standard tablet (iPad) and displayed on its screen. Three regions (i.e., D, E, and F) on the abdominal phantom were selected as the anchor points where the ultrasound probe stayed during the scanning (Fig. 12 (b)).

In the first experiment, region D was regarded as the target and the ultrasound probe was placed on it. This experiment aimed to acquire clear images of the spine, portal vein, vena cava, and aorta in region D. We stored 8N and 10N in the operating force database as the empirical operating forces for obtaining the ultrasound images of the target organs (Fig. 3). Based on the online adjustment method, the preliminary operating force was set to 8N and would be adjusted to 10N if no clearly visible image of the target area was obtained. During the procedures, the force control mechanism kept the operating force constant based on the CFGRD, and the force/torque measurement mechanism measured the operating forces and torques. The experiment had the same operation process as Fig. 4 (a). In the second experiment, according to the experimental results in the first experiment, we set 10 N as the ideal operating force for achieving various organs and tissues, and the ultrasound probe moved along regions D, E, and F. This experiment had the same operation process as Fig. 4 (b).

#### 2) Results and discussions

**Fig. 13:**
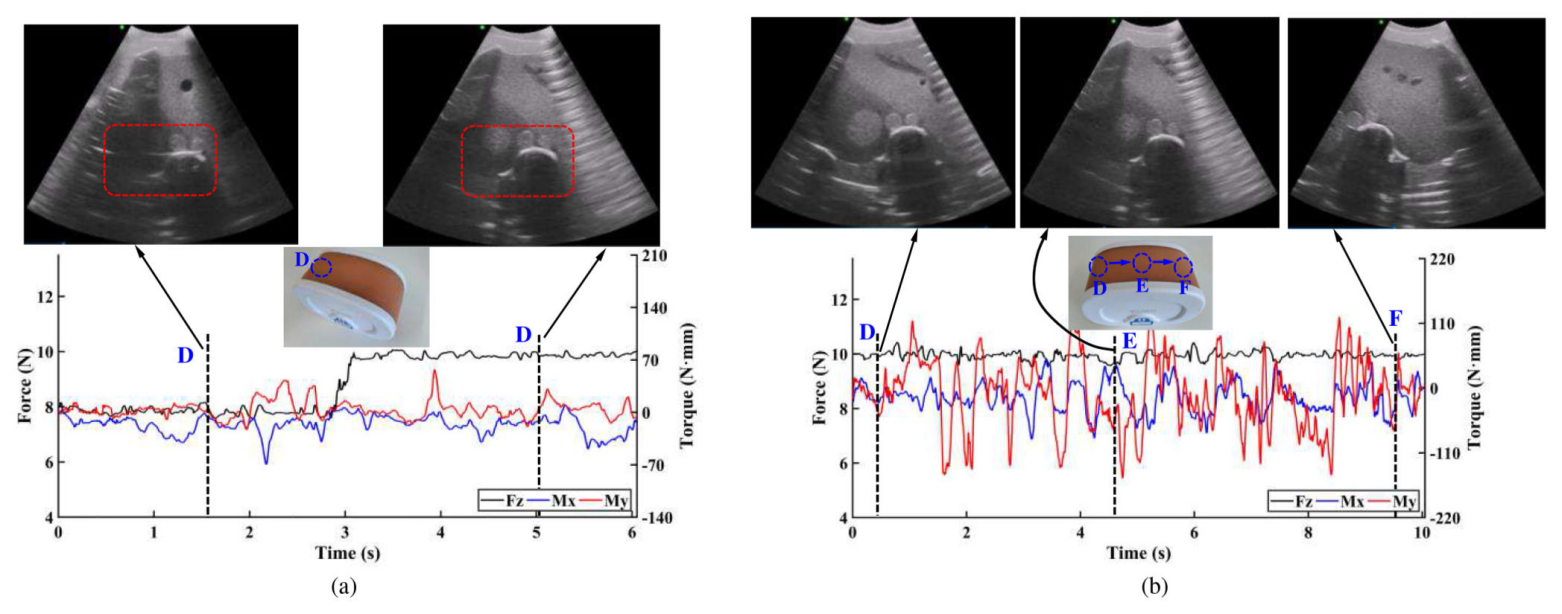
Operating forces/torques and acquired images in the first (a) and second (b) experiments.

The operating forces, torques, and images of various regions acquired in these two experiments are shown in Fig. 13. In Fig. 13 (a), the spine, portal vein, vena cava, and aorta in the acquired images (i.e., in the red dotted box) are not clearly visible with the operating force of 8 N; they become clearly visible when the scanning was performed with 10 N and a greater scanning depth was achieved. During the operation, the operating forces and torques were measured, and the black, blue, and red lines in the figure represent the forces in the *z*-direction, the torques around the *x*-direction, and the torques around the *y*-direction, respectively. The maximum error for generating constant force is 0.48 N; the maximum and minimum operating torques around the *x*-direction are 9.28 N·mm and -69.78 N·mm, respectively; the maximum and minimum operating torques around the *y*-direction are 57.02 N·mm and -23.14 N·mm, respectively. The operating force remained approximately constant due to the force control mechanism, while the operating torques varied with the operation.

In the second experiment, the operating force was maintained at 10 N during the operation, and images with various structures were obtained as the ultrasound probe slid over the abdominal phantom along regions D, E, and F (Fig. 13 (b)). The same operating force results in similar scanning depth, and thus it will be convenient for sonographers to find and observe target organs or tissues with similar depths. The maximum error for generating constant force is 0.57 N; the maximum and minimum operating torques around the *x*-direction are 47.71 N·mm and -86.51 N·mm, respectively; the maximum and minimum operating torques around the *y*-direction are 127.81 N·mm and -52.92 N·mm, respectively. The operating torques around the *y*-direction have greater amplitude and change frequency than those around the *x*-direction. This is mainly because the *y*-axis of the ultrasound probe is perpendicular to the moving direction (i.e., from region D to region F).

In these two experiments, since the operating forces could be kept constant and adjusted online based on the operating force database, the operator only needed to focus on observing ultrasound images and did not need to pay attention to the values of the operating forces and the contours of scanned areas. In other words, great operating forces introduced by accidental operations during the scanning can be eliminated. Moreover, the scanning depth was determined and would not change with the operation when the operating force was set as a constant value. Therefore, the operation safety and efficiency would be significantly improved. On the other hand, the operating force database with recommended operating forces for different targets facilitated the operators to find the targets quickly. In the experiments, operating forces of 8 N and 10 N were stored in the operating force database and regarded as the empirical operating forces. These values were determined based on operations on the commercial abdominal phantom rather than the clinical scanning. With respect to the operating force database for the actual scanning, it needs to be established based on real operation data and the sonographers’ professional experience. Additionally, the constant operating force would be helpful in decreasing patients’ discomforts introduced by undesired or great forces.

To be able to use the proposed online adjustment method and accumulate clinical data, sonographers can perform the preliminary setting of the operating force according to their professional experience to drive the online adjustment method, and during scannings, various operating forces for different tissues/organs will be recorded and stored in the database. Based on the accumulated operating forces, sub-databases can be built, which include the required operating forces for various tissues/organs of different patients. The sub-databases for different patients can be automatically selected by the robot via patients’ ages, fat thicknesses, physical conditions, and some other clinical indicators. However, a tremendous amount of clinical data will be needed and used as references or training samples. A trade-off is sonographers perform a force test on a specific region of the patients to select the sub-databases for patients before each scanning. For example, sonographers try to obtain images of a cross-section of the left side of the kidney and record the operating force when the images become clear. If the force is, for example, 7N, the patient will be determined as the type of patient with 7N; the type of patient is determined according to the measured operating force. The type of patient determines the type of sub-database, and thus the robot can complete scanning based on this sub-database.

## IV Conclusion

In this paper, a force/torque measurement mechanism and online adjustment method were proposed and integrated into the ultrasound robot with the force control mechanism. Calibration experiments were conducted to obtain the calibration parameters of the proposed force/torque measurement mechanism. Simulations and various experiments were carried out to evaluate the performance of the ultrasound robot. Experimental results show that the proposed ultrasound robot has the ability to provide an approximately constant operating force, measure the operating forces/torques, and adjust the operating force online to acquire ultrasound images of objects at various positions and with different depths. The proposed ultrasound robot has the potential to improve operation safety, continuity, and efficiency and decrease the discomfort of patients. However, there are still some limitations in this research. In the phantom experiments, we employed hypothetical data to replace the actual one and drive the proposed online adjustment method. The proposed online adjustment method is a clinical data-based approach and an operating force database needs to be established through actual scanning data. In addition, in the online adjustment method, the judgment of the image quality is currently determined by sonographers, and automatic image judgment can be employed by integrating our previous research from the iFIND project (https://www.ifindproject.com/) [30–32] in the future. Finally, the commercial abdominal phantom differs from the human abdomen in hardness, material, and structure, and thus volunteer studies are to be conducted in future work.

## Figures and Tables

**Table 1:** Characteristics Of The Force/Torque Measurement Mechanism

Type	F_z_	M_x_	M_y_
**Measurable Ranges**	±12 N	±168 N•mm	±168 N•mm
**Linearity**	0.9984	0.9978	0.9982
**Sensitivity**	0.456V/N	15V/(N•m)	15.01V/(N•m)
**Condition number**	———— 30.59 ———————		
